# Ultrasound-guided pulsed radiofrequency treatment of the cervical sympathetic chain for complex regional pain syndrome: A retrospective observational study

**DOI:** 10.1097/MD.0000000000005856

**Published:** 2017-01-10

**Authors:** Eung Don Kim, Woo Joo Yoo, Yoo Na Kim, Hue Jung Park

**Affiliations:** a Department of Anesthesiology and Pain Medicine, Daejeon St. Mary's Hospital, School of Medicine, The Catholic University of Korea, Seoul, Korea; b Department of Anesthesiology and Pain Medicine, Seoul St. Mary's Hospital, School of Medicine, The Catholic University of Korea, Seoul, Korea.

**Keywords:** cervical sympathetic chain, complex regional pain syndrome, pulsed radiofrequency, stellate ganglion block, ultrasound

## Abstract

The stellate ganglion is a common target to manage neuropathic pain in the upper extremities. However, the effect duration of a single stellate ganglion block is often temporary. To overcome the short-term effects of a single sympathetic block, pulsed radiofrequency (PRF) can be applied. The aim of the present study was to investigate the efficacy of PRF on the cervical sympathetic chain under ultrasound guidance for complex regional pain syndrome (CRPS).

Twelve CRPS patients who underwent PRF on the cervical sympathetic chain were enrolled in this retrospective analysis. Under ultrasound guidance, PRF was performed for 420 seconds at 42°C on the C6- and C7-level sympathetic chain.

The pain intensity decreased significantly at 1 week after the procedure. Overall, 91.7% of patients experienced at least moderate improvement. A positive correlation was observed between the extent of pain reduction at 1 week after PRF and the degree of overall benefit (*r* = 0.605, *P* = 0.037). This reduction in symptoms was maintained for a mean of 31.41 ± 26.07 days after PRF. There were no complications associated with this procedure.

PRF on the cervical sympathetic chain, which can be performed easily and safely under ultrasound guidance, should be considered an option for managing CRPS of the upper extremities.

## 1 Introduction

The stellate ganglion block (SGB) is widely used to manage neuropathic pain in the upper extremities.^[1]^ In 80% of the population, the stellate ganglion is formed by the fusion of the inferior cervical ganglion and first thoracic sympathetic ganglion and is located anteriorly to the neck of the first rib and extends to the interspace between the C7 and T1 vertebrae.^[2]^ It is difficult to access the true anatomical location of the stellate ganglion with a needle, and in approximately 90% of cases, the vertebral artery runs anteriorly at the C7 level and enters the foramen of the transverse process; therefore, blind SGB has traditionally been applied to the middle cervical sympathetic ganglions at the C6 level. However, in approximately 10% of the population, the vertebral artery may be exposed at the C6 level. In these individuals, blind SGB could result in catastrophic complications.^[3]^

More recently, the use of ultrasound guidance has allowed SGB to be performed without vascular or nerve injury.^[4]^ However, a single sympathetic block often provides only short-term effects. In addition, frequent procedures may result in complications such as infection or tissue or nerve injury. Complications in the cervical region can be particularly life-threatening.

To overcome the short-term nature of a single sympathetic block, methods such as a continuous catheter block of the sympathetic ganglions have been introduced.^[5–7]^ However, long-term catheterization generally requires hospitalization and may lead to infection. Alternative treatments include chemical neurolysis or thermal radiofrequency (RF), but these carry the risks of unnecessary complications such as irreversible nerve damage.^[8]^ Accordingly, these neurodestructive methods appear to be too risky for the treatment of cervical lesions.

Pulsed RF (PRF), a variation of thermal RF, has the advantage of causing little thermal injury as compared with RF.^[9,10]^ This advantage has led to the increasing application of PRF for the management of chronic pain.^[11]^ The electromagnetic field produced by the rapid electrical pulsation of PRF may have a biological effect on the target nerve. PRF induces an increase in c-fos expression and synaptic changes associated with transmission that are known as therapeutic mechanisms.^[11]^

To the best of our knowledge, only a few reports have discussed PRF treatment of the stellate ganglion.^[11–13]^ Furthermore, no studies have reported the application of PRF or RF of this region under ultrasound guidance for complex regional pain syndrome (CRPS).

In the present study, we performed PRF treatment of the cervical sympathetic chain at the levels of C6 and C7 under ultrasound guidance in patients with CRPS and retrospectively evaluated the efficacy of this procedure.

## 2 Methods

### 2.1 Participants

Permission for conducting this retrospective analysis was granted by the Institutional Ethics Committee of the Daejeon St. Mary's Hospital, Republic of Korea (DC15EISI0054). Twelve patients (10 men, 2 women, mean age: 45.16 ± 10.71 years) with diagnosed CRPS who were treated at the Pain Center of Daejeon St. Mary's Hospital were included in this study. These patients underwent PRF between February 2015 and May 2015. All diagnoses met the criteria for CRPS as recommended by the International Association for the Study of Pain^[14]^. The mean duration of symptoms was 4.58 ± 2.39 years. All patients had a history of trauma, and 8 patients underwent surgery before receiving a CRPS diagnosis (Table 1).

**Table 1:**
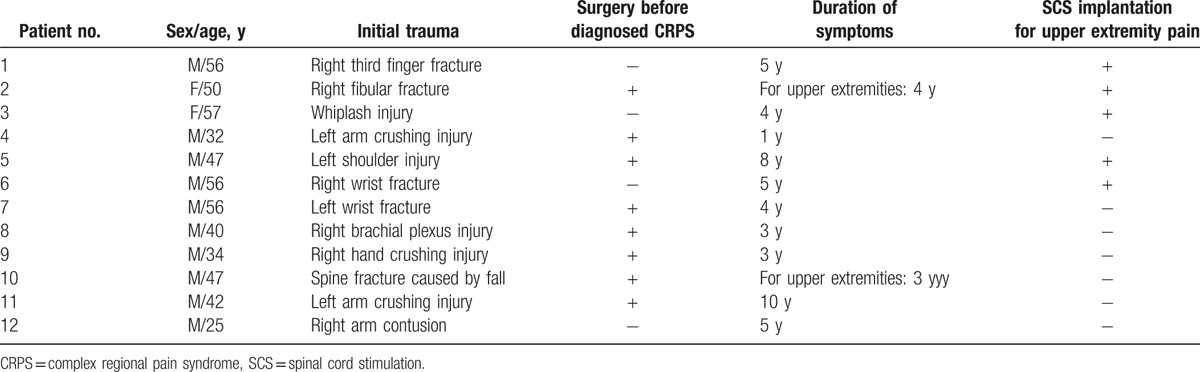
Demographic data and medical histories of the patients.

Various drugs such as anticonvulsants, tricyclic antidepressants, and opioids were used for symptom management in all patients. However, the effects of these medications were limited. Five patients had previously undergone spinal cord stimulation (SCS). Despite SCS implantation, these patients began to complain of neuropathic pain that measured 7 to 8 on a 10-point numeric rating scale (NRS) after a few months.

Multiple SGB procedures were performed under ultrasound guidance with 5 to 6 mL of 1% lidocaine. However, the effect of this treatment was generally extremely transient (i.e., in the range of a few hours). After confirming a positive response to SGB, PRF treatments of the cervical sympathetic chain at the level of C6 and C7 were performed to achieve a long-term analgesic effect. For cases with bilateral upper extremity pain, the procedure was performed on the side with more severe pain.

### 2.2 Procedure

**Figure 1:**
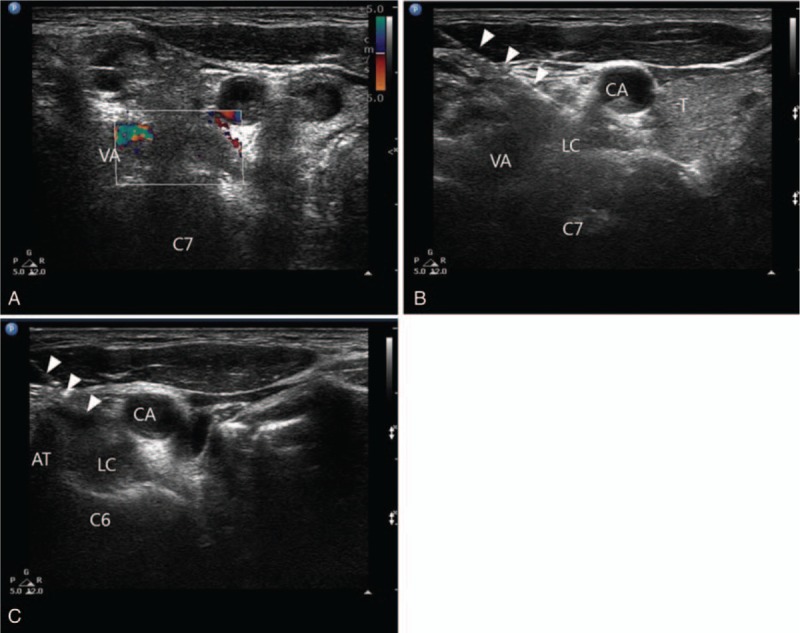
Ultrasound images of the C6 and C7 levels. (A) The vertebral and carotid arteries are identified in color Doppler mode at the C7 level. A needle was advanced under the prevertebral fascia on the surface of the longus colli muscle at the (B) C6 and (C) C7 levels. AT = anterior tubercle of the transverse process of C6, CA = carotid artery, LC = longus colli muscle, VA = vertebral artery. White arrowheads = needle.

**Figure 2:**
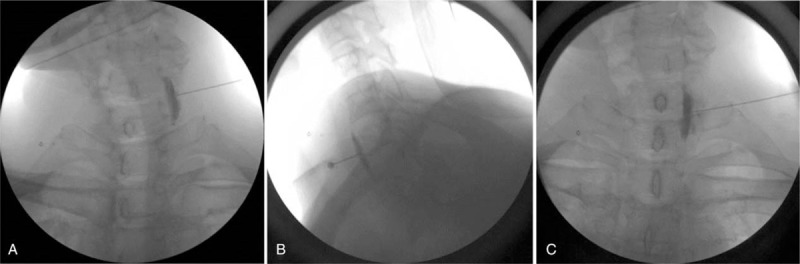
Fluoroscopic anteroposterior view of contrast spread at the (A) C6 level and (C) C7 level, and (B) a lateral view of the C6 level.

Each patient was placed in the supine position with the neck extended and the head rotated slightly to the opposite side. After preparing the skin with chlorhexadine, we confirmed the anterior tubercle of the transverse process of C6, the carotid artery, the internal jugular vein, and the longus colli muscle using a 5- to 12-MHz linear ultrasound transducer (Phillips Inc., Amsterdam, the Netherlands) with a short axial view. A color Doppler image was used to check the vessels through the needle course. After skin infiltration, a 5-cm long, 22-gauge RFK needle with a 5-mm active tip (Radionics Inc., Burlington, MA) was introduced in-plane from the lateral side of the probe. The needle tip was placed on the longus colli muscle and under the prevertebral fascia (Fig. 1B); subsequently, 0.2 mL of contrast agent was injected, and a fluoroscopic image was obtained to cross check for proper needle tip location (Fig. 2). Sensory and motor stimulation were applied using 50 and 2 Hz, respectively, and the patient was checked for paresthesia or phonation to exclude needle malpositioning. Next, a PRF was administered for 420 seconds at 42°C. Subsequently, the probe was moved in a more caudal direction, and the C7 level was confirmed by checking the disappearance of the anterior tubercle of the transverse process. The RF needle was then inserted carefully using an in-plane technique with real-time monitoring to avoid penetration of the pulsating vertebral artery (Fig. 1A, C). PRF was applied at the C7 level in the same manner as at the C6 level. After confirming negative blood aspiration, we injected 6 mL of 1% lidocaine through the needle and terminated the procedure.

### 2.3 Outcome measures

Temperature was measured by touch thermometer (patient monitor VM8; Phillips Inc.) at the volar aspect of both hands before and after the procedure. The NRS scores of participants were also evaluated before and immediately after the procedure and again at 1 week after the procedure. The duration of the effects of PRF on the cervical sympathetic chain was also investigated.

To investigate the overall benefits of this procedure, we surveyed the patients to determine the degree of benefit (%) once the PRF effect wore off. The patients were subsequently divided, according to the self-described degree of benefit, into groups with substantial (≥50%), moderate (≥30%), and minimal (<30%) improvement.^[15]^

### 2.4 Data analysis

Data are presented as means ± standard deviations for continuous variables. Data normality was evaluated using the Shapiro–Wilk test. In the present study, the pre- and post-procedure NRS scores were compared using the Wilcoxon signed-rank test. Correlations between variables were analyzed using Pearson coefficient of correlation. All data were analyzed using SPSS version 18.0 (SPSS Inc., Chicago, IL), and a *P* value less than 0.05 was considered statistically significant.

## 3 Results

After the procedure, a mean temperature difference of 1.39 ± 0.96°C was observed between the both hands. All patients reported pain reduction of ≥50% immediately after the procedure. Ten of the twelve patients (83.3%) reported varying degrees of analgesic effects at 1 week after the procedure. Two patients (17.7%) reported no prolonged pain reduction at 1 week, although 1 of these patients reported a moderate degree of overall improvement. The mean effect duration of PRF was 32.00 ± 25.55 days (Table 2).

**Table 2:**
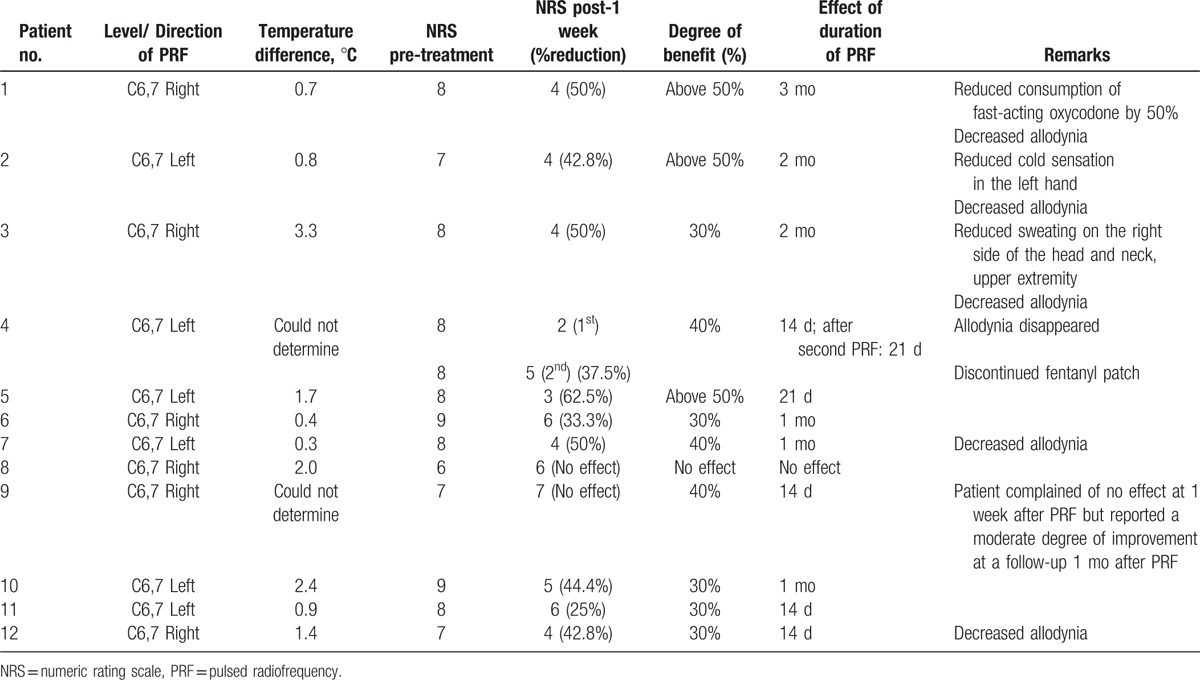
Clinical outcomes of pulsed radiofrequency treatment.

Allodynia either disappeared or decreased in 6 patients. The mean NRS scores were 7.75 ± 0.87 at baseline and 4.83 ± 1.19 at 1 week after the PRF procedure, and this reduction in NRS was statistically significant (*P* = 0.0005) (Table 3).

**Table 3:**
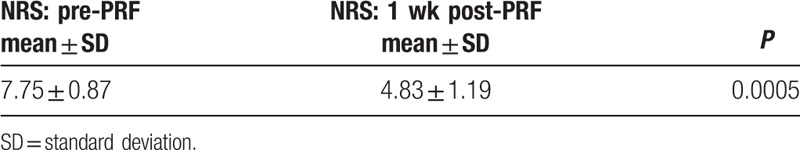
Changes in the numerical rating scale (NRS) score from before to 1 week after pulsed radiofrequency (PRF).

Regarding patient stratification, 25% of our study participants (n = 3) comprised the substantial improvement group. After including the moderate improvement group (n = 8, 66.7%), we noted that positive clinical benefits were achieved by 91.7% of patients.

**Figure 3:**
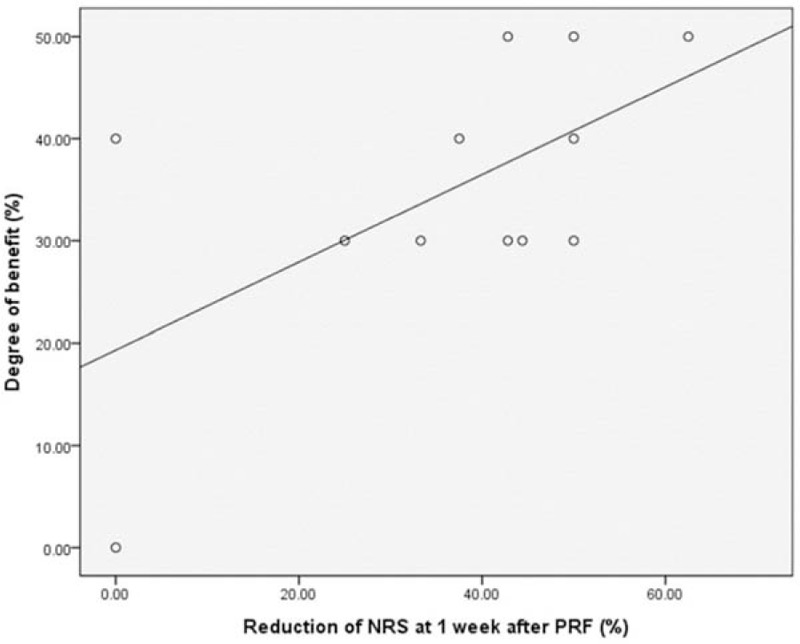
Scatter plot demonstrating a clear correlation between numeric rating scale (NRS) score reduction at 1 week after pulsed radiofrequency (PRF) and the overall degree of benefit.

The degree of NRS reduction at 1 week after PRF correlated positively with the overall degree of the benefit derived from the PRF (*r* = 0.605, *P* = 0.037) (Fig. 3).

No intravascular or epidural contrast patterns on fluoroscopic images and no severe complications, such as infection or hematoma formation, were observed. PRF did not have any effect on the existing SCS.

## 4 Discussion

In the present analysis, the majority of our patients reported positive results from PRF on the cervical sympathetic chain. Short-term outcomes such as NRS reduction at 1 week after the procedure generally correlated with the overall degree of each patient's satisfaction. One patient complained of a negative effect at 1 week after PRF. Interestingly, the same patient reported a moderate degree of improvement at 1 month after PRF. We cannot explain this delayed analgesic effect of PRF. However, this result might reconfirm that the mechanism of PRF is distinct from that of nerve blocks involving local anesthetics.

When applying thermal RF, the thermocoagulation effect is the strongest on both sides of the noninsulated needle tip.^[16]^ However, when RF is performed using a blind technique or with fluoroscopic guidance, the active needle tip and the cervical sympathetic chain are perpendicular to each other, rather than parallel. As a result, unnecessary tissue damage may occur around the active needle tip, rather than accurate target nerve lesioning in cases involving thermal RF at a relatively perpendicular angle.

The electromagnetic field density is greatest at the distal needle tip.^[16]^ Therefore, when performing PRF, the ideal needle placement is perpendicular to the target nerve. When the needle is advanced to the cervical sympathetic chain with ultrasound guidance using the in-plane technique, the angle between needle and targeted sympathetic chain should be relatively perpendicular rather than parallel (Fig. 1). In this respect, PRF might be a more appropriate option for the lesioning cervical sympathetic chain.

In the majority of studies involving RF or PRF treatment of the cervical sympathetic chain,^[10–12]^ the procedure was performed under fluoroscopic guidance. Fluoroscopy can, of course, confirm an accurate needle tip position, but cannot ensure vascular or nerve safety while advancing the needle.

**Figure 4:**
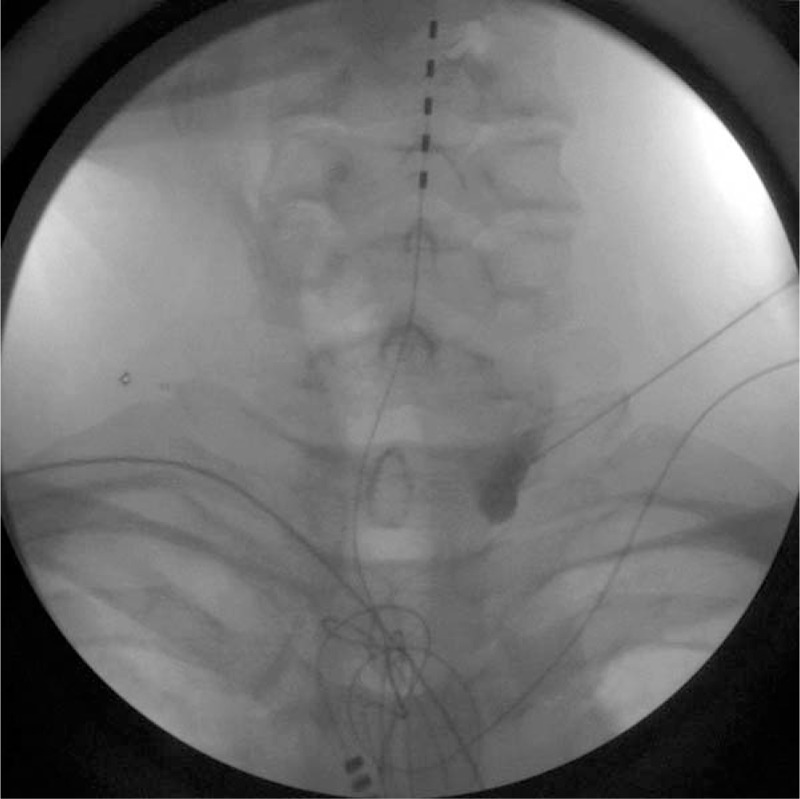
The needle tip is located at the T1 level, which is more adjacent to the anatomical location of the stellate ganglion. This patient had previously undergone implantation for spinal cord stimulation.

Ultrasound guidance allows real-time visualization of the vessels or pleura and thus helps to avoid injuring these structures. Therefore, even if the transducer moved more caudally below the C7 level, the needle could be advanced without injuring vulnerable structure and could reach closer to the true anatomical location of the stellate ganglion. This proximity to the true anatomical location is a strong advantage of using ultrasound. In the present study, although we did not intend for the needle tip to locate below the C7 level, in some cases, we could easily place the needle tip between C7 and T1, as confirmed using fluoroscopy (Fig. 4).

CT-guided RF of the stellate ganglion has also been introduced.^[17]^ CT guidance may facilitate accurate and safe needle placement. However, given the cost-effectiveness and convenience of the procedure and, above all, the advantage of the real-time monitoring of vulnerable structures, we believe that ultrasound guidance is more suitable in a clinical setting.

The range of effects associated with RF or PRF is much smaller than that of spreading a few milliliters of local anesthetic agent. This point could explain why some subjects in this study experienced negative effects with PRF after exhibiting positive responses to a single SGB, as well as the inconsistency of the PRF effects observed in this study.

Further technical development might be needed to accurately distinguish the sympathetic chain under ultrasound guidance. Another type of PRF, such as the bipolar mode, might also help to overcome the limited range of effects provided by PRF with a single-needle electrode.

In conclusion, using ultrasound-guided PRF on the cervical sympathetic chain, we achieved desirable outcomes in patients with upper extremity CRPS. This is the first study regarding PRF on the cervical sympathetic chain under ultrasound guidance in patients with CRPS of the upper extremities. PRF on the cervical sympathetic chain therefore appears to be a valid option for the management of CRPS of the upper extremities, and the incorporation of ultrasound can increase the ease and safety of this procedure. Undertaking a well-designed, prospective study with a large sample size in the near future is highly recommended.

### 4.1 Uncited reference

^[14]^.
